# The Recipient Vessel Hemodynamic Features Affect the Occurrence of Cerebral Edema in Moyamoya Disease After Surgical Revascularization: A Single-Center Retrospective Study

**DOI:** 10.3389/fneur.2022.890126

**Published:** 2022-05-16

**Authors:** Liang Xu, Yin Li, Yun Tong, Jun-wen Hu, Xu-chao He, Xiong-jie Fu, Guo-Yang Zhou, Yang Cao, Xiao-bo Yu, Hang Zhou, Chao-ran Xu, Lin Wang

**Affiliations:** Department of Neurosurgery, School of Medicine, The Second Affiliated Hospital, Zhejiang University, Hangzhou, China

**Keywords:** moyamoya disease (MMD), cerebral edema, recipient vessels, digital subtraction angiography (DSA), flow direction, stroke

## Abstract

**Objective:**

In moyamoya disease (MMD) with direct or combined revascularization, the initially hemodynamic recipient features are likely one of the main causes of acute hemodynamic disruption. Previous studies have explored the relationship between recipient diameter or flow velocity and postoperative complications, but there are still no optimal selection criteria with multiple potential recipient vessels. Cerebral edema is one of the most common radiological manifestations in the acute postoperative period. This study assessed the hemodynamic characteristics of cortex vessels related to postoperative cerebral edema.

**Methods:**

All patients who had undergone direct or combined revascularization with preoperative digital subtraction angiography (DSA) between 2019 and 2021 were eligible for inclusion in this study. The application of DSA was performed and regular radiological examinations were employed after surgery. DSA was analyzed with the hemodynamic features within chosen recipient vessels. Cerebral edema was identified as a low-density image on CT or high signaling in the MRI T2 phase. The recipient hemodynamic characteristics and demographic presentation, as well as clinical data, were retrospectively analyzed in this study.

**Results:**

A total of 103 patients underwent direct or combined revascularization with preoperative DSA. The mean age of this enrolled cohort was 44.31 ± 10.386 years, in which bilaterally involved MMD accounted for the main part. The preliminary correlation analysis found preoperative disease period (*p* = 0.078), recipients observed in angiography (*p* = 0.002), and surgery on the left (*p* = 0.097) may be associated with cerebral edema. The following regression analysis confirmed low occurrence of cerebral edema was accompanied by recipients observed in angiography (*p* = 0.003). After subdividing by flow direction and hemodynamic sources, the incidence rate of anterograde direction, anterior sources, and posterior sources were significantly lower than undetected recipients.

**Conclusions:**

Cerebral edema is a common radiological manifestation in MMDs after surgery. In this study, the observation in angiography reliably identifies a variety of physiological or pathological recipient detection, flow direction, and hemodynamic sources in patients with MMD after revascularization, which indicates the selection strategy of potential recipients and highlights the importance of recipient observability in DSA. Meanwhile, vascular conditions determined by recipient hemodynamics meditate the occurrence of postoperative cerebral edema.

## Introduction

Moyamoya disease (MMD) is a chronic cerebrovascular disease that is characterized by progressive steno-occlusive in bilateral internal carotid arteries and their proximal branches (1). Surgical revascularization has been recognized as an effective treatment to improve the impaired cerebral hemodynamics and decreased the risk of recurrent stroke (2). However, a previous study found about 16% of MMDs suffered a postoperative stroke with permanent deficits (3) and the watered-shift phenomenon was as high as 10.9% after surgery (4). Surgical revascularization changed the distribution of the recipient's vessels and the sharp hemodynamic shift caused by vascular anastomosis may boost postoperative neurological morbidity. Recent research demonstrated that blood flow changes before and after vascular anastomosis were evident in MMDs with postoperative complications (5). Recipients with an initially retrograde flow direction presented great potential for flow increase after surgery (6) and arteries with the earliest intraoperative fluorescence emission were prone to occur symptomatic complications (7). In addition, hemodynamic sources of recipient arteries were concerned with postoperative hyperperfusion (8).

The radiologic incidence of stroke was higher than symptomatic stroke, in which 33.3% of MMDs were reported with postoperative diffusion-weighted imaging (DWI)-detected lesions (9). Cerebral edema is a common imaging performance related to hemodynamic compromise after surgical revascularization and may be warning sign that focal parenchymal tissues fail to adapt to hemodynamics disruption. Thus, this study aimed to analyze the effects of recipient hemodynamic features on postoperative cerebral edema in MMDs.

## Materials and Methods

### Patients Selection

**Figure 1:**
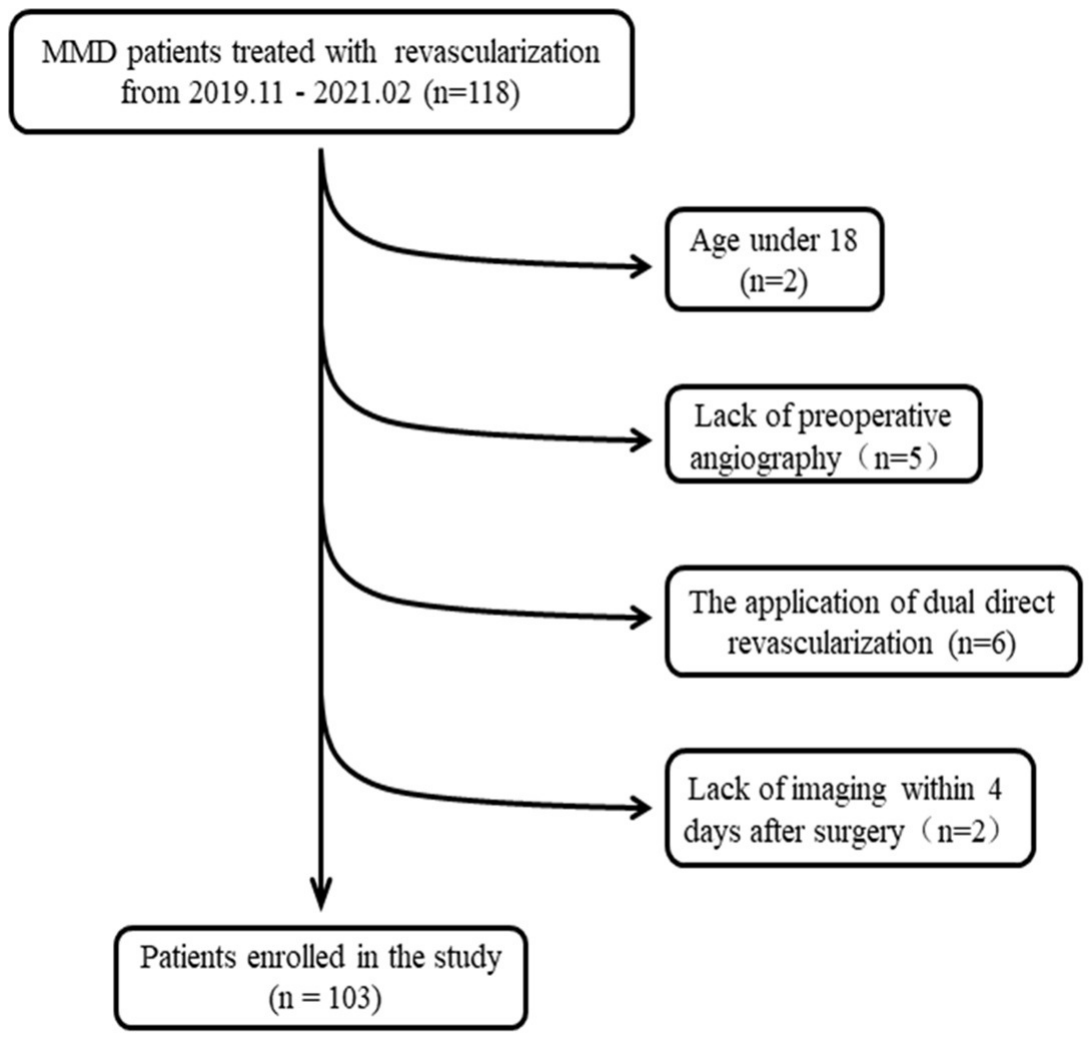
Flow diagram for the study selection process.

This study included 103 MMDs with surgical revascularization at our hospital between November 2019 and February 2021 after 15 patients were excluded (Figure 1). The diagnosis of MMD was confirmed based on the guidelines for MMD diagnosis and treatment published by the Research Committee on the Pathology and Treatment of Spontaneous Occlusion of the Circle of Willis (10). Besides, the inclusion criteria of this study were as follows:

(1) Age ≥ 18 years;
(2) Definite diagnosis of MMD confirmed by DSA;
(3) The application of direct or combined revascularization;
(4) Radiological examinations, including computed tomography (CT) or magnetic resonance imaging (MRI), were performed within 4 days after surgery.

The exclusion criteria were as follows:

(1) The employment of dual direct revascularization;
(2) Lack of preoperative angiography in our hospital.

### Surgical Procedures

Direct revascularization indicates superficial temporal artery (STA)—middle cerebral artery (MCA) bypass (11). Combined revascularization has been described previously (12) and consists of direct and indirect revascularization (encephalo-duro-myo-synangiosis, EDMS). Meanwhile, intraoperative indocyanine green was immediately injected to confirm the patency of anastomotic sites.

### The Evaluation of Recipient Hemodynamic Features and Postoperative Cerebral Edema

The hemodynamic characteristics of the recipient's were assessed through preoperative DSA by two experienced neurosurgeons. Of note, the flow direction of the recipient's vessels was categorized as follows:

(1) Anterograde direction: blood flow from M3 segment to M4 segment;
(2) Retrograde direction: blood flow from M4 segment to M3 segment;
(3) Undetected direction: no or little flow direction was detected in angiography.

The hemodynamic sources of the recipient's vessels were categorized as follows:

(1) Anterior sources: blood supply was from an anterior cerebral artery (ACA) or MCA;
(2) Posterior sources: blood supply was from a posterior cerebral artery (PCA) or vertebrobasilar artery;
(3) Undetected sources: no or few hemodynamic sources were detected in angiography.

**Figure 2:**
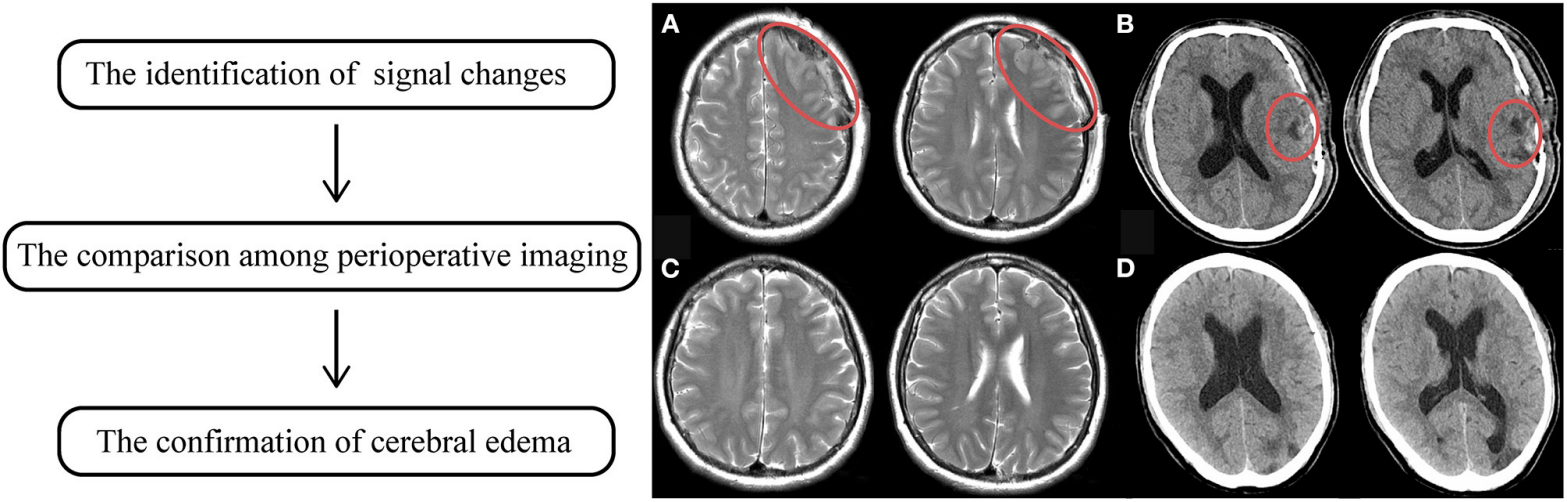
The flowchart of the identification of cerebral edema. The left indicates the steps of identification. **(A)** showed high signaling changes in MRI T2 phase. **(B)** showed low density changes in CT, in which the red circle manifested altered regions. **(C,D)** was preoperative radiological sequences of **(A,B)**, respectively. It was proven that those signaling changes just occurred after surgery.

The evaluation of cerebral edema was relatively fussy. The first step is to identify signal changes around the anastomotic area of postoperative radiological examinations, which presented as either slightly low-density on CT or high-signaling in the MRI T2 phase. Subsequently, the comparison among perioperative imaging was applied to confirm whether the changes were recent, which means occurring or not. The occurrence of cerebral edema was confirmed by two experienced neurosurgeons independently and the results were reached after consensus, as shown in Figure 2.

### Data Analysis

The SPSS 24.0 (SPSS, Inc., Chicago, IL, USA) was used for statistical analysis. A descriptive summary is presented as mean ± standard deviation (SD), median (interquartile range), frequency, or percentage, as appropriate. For comparison between the non-postoperative cerebral edema group and postoperative cerebral edema group, an independent-sample *t*-test, Pearson's chi-squared, or Fisher's exact test correlation analysis was performed. Afterward, the logistic regression analysis was conducted for significant factors achieving *P* < 0.10 in the univariate analysis. All tests were two-tailed. *P* < 0.05 was considered to be statistically significant.

## Results

### Demographics and Clinical Presentation

Of the 118 consecutive patients with MMD, who underwent surgical revascularization from November 2019 to February 2021, we identified 103 MMDs who met the inclusion and exclusion criteria of this research (Figure 1). The mean age of MMDs was 44.31 ± 10.386 years. The female ratio was 69.9% in this study. Eighty-three out of 103 (80.6%) patients presented with bilateral MMD. Thirty-four percent of patients presented with postoperative cerebral edema. The other clinical characteristics of total MMDs are summarized in Table 1.

**Table 1:** The baseline characteristics of enrolled MMD patients.

	**Patients (*n* = 103)**
Age, yrs	44.31 ± 10.386
Female (%)	73 (69.9)
**Comorbidity (%)**
Smoking	19 (18.4)
Alcohol consumption	20 (19.4)
Dyslipidemia	24 (23.3)
Hypertension	33 (32.0)
Diabetes	10 (9.7)
**MMD characteristics (%)**
Bilateral involved	83 (80.6)
Onset manifestation	
Ischemic	58 (56.3)
Hemorrhagic	39 (37.9)
Others	6 (5.8)
Preop disease period, mos^*^	5.00 (10)
Suzuki stage	
1	13 (12.6)
2	18 (17.5)
3	52 (50.5)
4	18 (17.5)
5	2 (1.9)
**Surgical information (%)**
Surgical type	
Direct revascularization	7 (6.8)
Combined revascularization	96 (93.2)
The left side of operation	53 (51.5)
Postop cerebral edema (%)	35 (34.0)
mRS on admission^*^	0.00 (0)

*MMD, moyamoya disease; Preop, preoperative; Postop, postoperative.*

* *Indicates that values are presented as median (interquartile range)*.

### Effects of Recipient Vessel Characteristics on Cerebral Edema

Initially, we analyzed basic features between the non-postoperative (non-postop) edema group and the postoperative (postop) edema group. As shown in Table 2, the demographics of the two groups were similar and had no statistical significance. And the comorbidities apart from MMD in groups presented no significant differences. Besides, disease-related aspects were consistent between the two groups. However, the preoperative disease period was inclined to be shorter in the postop edema group than the non-postop edema group (*p* = 0.078). Remarkably, the proportion of recipient's vessels observed in preoperative angiography was less in the postop edema group significantly, compared with the non-postop edema group (*p* = 0.002), which presented as 54.3 vs. 82.4%. Meanwhile, the duration of the postop edema group was prone to be longer than the non-postop edema group statistically (17.94 ± 7.840 vs. 14.66 ± 3.896, *p* = 0.024). Also, the time of postoperative imaging was irrelevant to cerebral edema (*p* = 0.181). After possible factors were included in logistic regression, we found recipient vessels observed in preoperative angiography were an independent factor associated with postoperative cerebral edema (OR = 3.930, 95% CI: 1.579–9.778, *p* = 0.003).

**Table 2:** The factors related with postoperative cerebral edema in adults with moyamoya.

	**Univariate analysis**			**Multivariate analysis**		
	**Non-postop edema (*n* = 68)**	**Postop edema (*n* = 35)**	***p*-value**	**OR**	**95% CI**	***p*-value**
Age, yrs	43.66 ± 10.633	45.57 ± 9.915	0.379^a^			
Female (%)	49 (72.1)	23 (65.7)	0.506^b^			
Smoking (%)	12 (17.6)	7 (20.0)	0.771^b^			
Bilateral disease (%)	52 (76.5)	31 (88.6)	0.141^b^			
Onset manifestation (%)			0.407^b^			
Ischemic	37 (54.4)	21 (60.0)				
Hemorrhagic	24 (35.3)	13 (37.1)				
Others	7 (10.3)	1 (2.9)				
Preop disease period, mos	10.61 ± 16.137	6.71 ± 5.800	0.078^a^			
Suzuki stage (%)			0.905^c^			
1	8 (11.8)	5 (14.3)				
2	11 (16.2)	7 (20.0)				
3	35 (51.5)	17 (48.6)				
4	13 (19.1)	5 (14.3)				
5	1 (1.5)	1 (2.9)				
Recipient vessels observed in preop angiography (%)	56 (82.4)	19 (54.3)	0.002^b^	3.930	1.579–9.778	0.003
Surgery on the left (%)	31 (45.6)	22 (62.9)	0.097^b^			
Time of postop imaging, days	2.37 ± 0.976	2.66 ± 1.136	0.181^a^			
Duration, days	14.66 ± 3.896	17.94 ± 7.840	0.024^a^			

*Preop, preoperative; postop, postoperative.*

a *Independent-samples t-test.*

b *Pearson chi-square.*

c *Fisher's exact test*.

**Figure 3:**
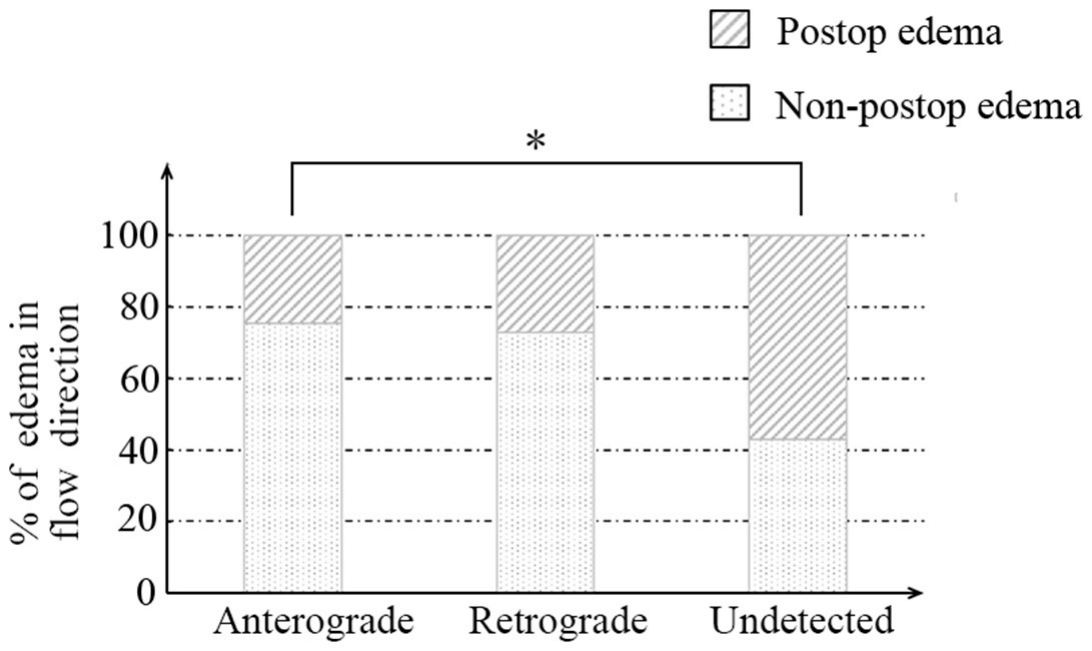
The relationship between recipient flow direction and postoperative cerebral edema. * indicates *p* < 0.05.

**Figure 4:**
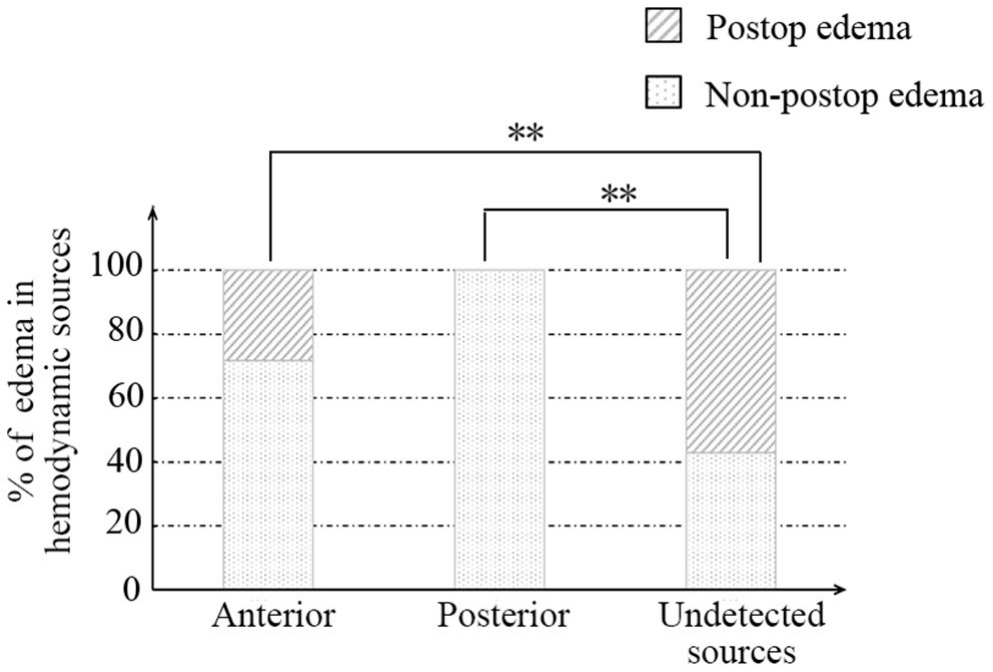
The relationship between recipient hemodynamic sources and postoperative cerebral edema. ** indicates *p* < 0.01.

Afterward, we further investigated the flow direction and hemodynamic sources of recipient vessels on the occurrence of cerebral edema. The two different features had obvious statistical differences in the two groups (*p* = 0.012; *p* = 0.002, Table 3). Of note, the interrater reliability between the evaluation of two independent surgeons in the flow direction, hemodynamic sources, and cerebral edema is a significantly almost perfect agreement (*K* = 0.906; *K* = 0.903; *K* = 0.916, Table 4). On the one hand, the occurrence rate of cerebral edema in the undetected direction was higher than anterograde direction (*p* = 0.004, Figure 3), but no significant differences were shown between the retrograde direction and the undetected direction. On the other hand, undetected sources were vulnerable to cerebral edema statistically, compared with anterior sources and posterior sources (*p* = 0.008; *p* = 0.005; Figure 4).

**Table 3:** The hemodynamic features of recipient vessels involved with postoperative cerebral edema.

**Preop recipient vessels characteristics (%)**	**Non-postop edema (*n* = 68)**	**Postop edema (*n* = 35)**	***p*-value**
Observed in angiography	56 (82.4)	19 (54.3)	0.002
Flow direction (Ref = undetected)			0.012^a^
Anterograde	40 (58.8)	13 (37.1)	0.004^b^
Retrograde	16 (23.5)	6 (17.1)	
Undetected	12 (17.6)	16 (45.7)	
Hemodynamic sources (Ref = Undetected sources)			0.002^a^
Anterior	48 (70.6)	19 (54.3)	0.008^b^
Posterior	8 (11.8)	0 (0.0)	0.005^a^
Undetected sources	12 (17.6)	16 (45.7)	

*Preop, preoperative; postop, postoperative.*

a *Fisher's exact test.*

b *Pearson chi-square*.

**Table 4:** The interrater reliability involved in the estimation of recipient hemodynamic features and postoperative cerebral edema.

	**Surgeon 1**	**Surgeon 2**	***p*-value**	**Kappa (95% CI)**
Flow direction (%)			<0.001	0.906 (0.833, 0.979)
Anterograde	51 (49.5)	55 (53.4)		
Retrograde	24 (23.3)	20 (19.4)		
Undetected	28 (27.2)	28 (27.2)		
Hemodynamic sources (%)			<0.001	0.903 (0.821, 0.985)
Anterior	65 (63.1)	68 (66.0)		
Posterior	28 (27.2)	28 (27.2)		
Undetected sources	10 (9.7)	7 (6.8)		
Cerebral edema (%)			<0.001	0.916 (0.836, 0.996)
Edema	36 (35.0)	38 (36.9)		
Non-edema	67 (65.0)	65 (63.1)		

## Discussion

In this study, 103 patients accepted surgical revascularization and ischemia was the major onset manifestation in enrolled MMDs. Disease severity was classified by the angiography-based Suzuki classification and stage 3 accounted for the bulk of all patients. No severe neurological deficits were observed on admission. Thirty-five cases were caught in cerebral edema. The anastomosis to undetected recipient vessels in angiography was inclined to cause postoperative edema. After further subdivision of recipient hemodynamic features, the anterograde direction was less prone to develop edema. Meanwhile, the incidence of edema in anterior and posterior hemodynamic sources was lower than that in recipient vessels with undetected sources, respectively.

Surgical revascularization has been applied in the treatment and effectively improves the long-term outcome of MMD (13, 14). Direct revascularization immediately improves impaired hemodynamic status after surgery, but ischemic parenchymal tissues are possibly unadaptable to dramatic hemodynamic changes. There were 16–20% of patients involved with complications after direct or combined revascularization (2, 3), which was higher than other craniotomy surgery. The radiological signal changes are before the onset of neurological symptoms. A previous study reported that 27.5% MMDs after STA-MCA bypass experienced cerebral hyperperfusion syndrome (15). Radiological hyperperfusion syndrome is defined as a great increase in recipient cerebral blood flow (16), which requires specific sequences measuring hemodynamic differences. As a postoperative common radiological appearance, the discrimination of cerebral edema requires simple image examinations. Surgical techniques caused little disturbance and limited edema on the cerebral cortex, while the hemodynamic compromise occurred in the blood flow competition between the donor and recipient arteries (17). As the watershed shift, reversed flow direction may contribute to the hypoperfusion of the distal MCA field (18), followed with the appearance and progression of cerebral edema. Consequently, edema after bypass probably implied the inadequate adaptation of focal brain tissues in early postoperative hemodynamic variation.

The selection of recipient vessels is likely one of the main determinants in the anastomotic procedure (6, 19). The most common strategy is the recipient with the largest diameter, which seems to guarantee the favorable anastomotic process and draw high compensatory outflow as a primary determinant of direct revascularization function (20). However, the matched calibers between STA and MCA are sufficient to avoid anastomotic failure (19). Also, hemodynamic features of recipient's vessels are heavily associated with postoperative complications. Multiple applications have focused on recipient vessel selection to reduce acute complications *via* intraoperative indocyanine green video angiography, which indicated the earliest emission was prone to occur symptomatic hyperperfusion, and the latest was related to white thrombus of anastomotic sites (5, 7, 21).

Recipient vessels with preoperative detection in DSA are at a significantly lower risk of cerebral edema, as shown in Table 2. The angiographic appearance of recipient's vessels manifests the strong communication between cortex vessels and large intracranial arteries, which also indicates the potential adaptation of neighboring parenchymal tissues. In contrast, the missing angiographic recipients presented high rates of edema. Undetected angiographic vessels were accompanied by high vascular resistance or severe proximal perfusion deficit. After the subsequent blood flow shock from donor arteries, cortical vessels showed maladaptive manifestations, of which the most familiar is cerebral edema.

**Figure 5:**
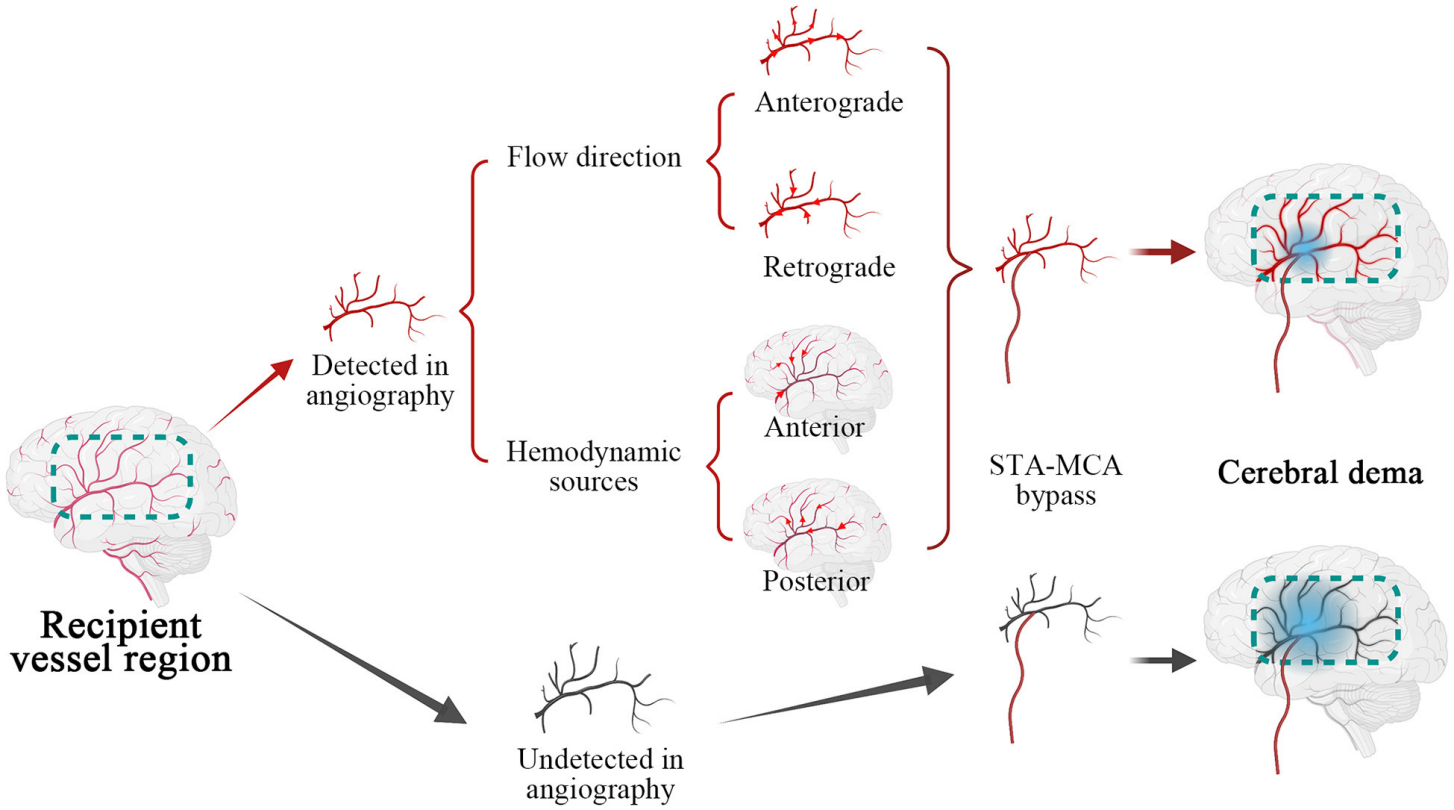
Illustrations of recipient hemodynamic features on the cerebral edema. Marked by the dotted box in green, recipient vessel region was categorized as angiographic detected in red and undetected in black. The cortex vessels were applied with superficial temporal artery (STA)- middle cerebral artery (MCA) bypass, after subdivided by flow direction and hemodynamic sources, in which red arrows indicated specific directions of blood flow. Subsequently, the recipients with different hemodynamic characteristics developed cerebral edema implied by gradient blue circle and undetected recipients were prone to postoperative cerebral edema.

Furthermore, an initially anterograde blood flow direction, anterior and posterior hemodynamic sources within possible recipients, was less likely to occur in cerebral edema than undetected flow direction, shown in Figures 3, 4 and Table 3. We believe that these characteristics indicate great accommodated capacity with little vascular resistance because failure to show in DSA is caused by arterial steal and loss of vascular autoregulation. In general, the primary prerequisite of initially regular flow orientations requires integrated vascular networks. Besides, the disposition of extracranial strike recruits local strong vascular autoregulation. Similarly, anterior or posterior hemodynamic sources within potential cortex vessels could indicate the integrality of the vascular bed. Intriguingly, the combination of heel and toe recipient orientations occasionally appeared in the anterior or posterior hemodynamic sources. In effect, the blood supply from anterior or posterior cerebral arteries reverses through distal cortical vessels and into the recipient encephalic region, in a certain area which run in forward directions at times, shown in Figure 5. The two different categories seem to be arguable in position-specific flow orientations, but there are exactly two diverse directions in particular arteries. With these mentioned discoveries, we have ample reason to believe cortex vessels with anterior directions from anterior or posterior hemodynamic sources could achieve less cerebral edema. Meanwhile, the specious contradiction may also support the hypothesis that the distribution dynamics of preceding DSA through intracranial braches and into the recipient bed serve as an indicator of vascular conditions. After all, complementary flow from the fish-mouthed graft to promote the filling of the recipients requires sufficient vascular spaces for shock absorption in the acute postoperative period (22). It is not a question of which direction or source of recipient's vessels are, but rather how much of recipients could be detected in angiography. Thus, the recipient observation in DSA indicates vascular conditions and serves as a mediator in cerebral edema. Of note, the medical strategy in enrolled MMDs after surgery reduced changes in blood flow requiring receptor vascular adaptation. The unstable blood pressure possibly caused sharp changes in extra-intracranial arterial pressure difference. The strict control of blood pressure between 120 and 140 mmHg was beneficial to narrow down hemodynamic fluctuations and avoid other fatal complications, such as anastomotic rupture or bleeding.

Several potential limitations of this study should be noted. First, it was a retrospective analysis with small sample size. The operative indications and surgical procedures were based on individual characteristics and institutional experience. Second, cerebral edema was simply recognized by daily CT or MRI examinations, and there were still patients with mild cerebral edema missing in this study. Third, further multicenter studies with a larger cohort of MMD and focused on the type of edema and risk factors should be conducted.

## Conclusions

Cerebral edema is a common radiological manifestation in patients with MMD after surgery. In this study, the observation in angiography reliably identifies a variety of physiological or pathological recipient detection, flow direction, and hemodynamic sources in patients with MMD after revascularization, which indicates the selection strategy of potential recipients and highlights the importance of recipient observability in DSA. Meanwhile, vascular conditions determined by recipient hemodynamics meditate the occurrence of postoperative cerebral edema.

## Data Availability Statement

The raw data supporting the conclusions of this article will be made available by the authors, without undue reservation.

## Ethics Statement

The studies involving human participants were reviewed and approved by the Local Ethics Committee of The Second Affiliated Hospital of School of Medicine, Zhejiang University. The patients/participants provided their written informed consent to participate in this study.

## Author Contributions

Material preparation, data collection, and analysis were performed by LX and YL. The first draft of the manuscript was written by LX and all authors commented on previous versions of the manuscript. All authors contributed to the study conception, design, read and approved the final manuscript.

## Funding

This work was supported by the National Science Foundation of China (Nos. 81870910 and 82171271) and Zhejiang Provincial Natural Science Foundation of China (LQ19H160039).

## Conflict of Interest

The authors declare that the research was conducted in the absence of any commercial or financial relationships that could be construed as a potential conflict of interest.

## Publisher's Note

All claims expressed in this article are solely those of the authors and do not necessarily represent those of their affiliated organizations, or those of the publisher, the editors and the reviewers. Any product that may be evaluated in this article, or claim that may be made by its manufacturer, is not guaranteed or endorsed by the publisher.

## Abbreviations

ACA: Anterior cerebral artery
CT: Computed Tomography
DSA: Digital Subtraction Angiography
MCA: Middle cerebral artery
MMD: Moyamoya disease
STA: Superficial temporal artery.
